# Exploring the Relationship Between Usage and Outcomes of an Internet-Based Intervention for Individuals With Depressive Symptoms: Secondary Analysis of Data From a Randomized Controlled Trial

**DOI:** 10.2196/12775

**Published:** 2019-08-01

**Authors:** Angel Enrique, Jorge E Palacios, Holly Ryan, Derek Richards

**Affiliations:** 1 E-mental Health Research Group School of Psychology Dublin Ireland; 2 Clinical Research & Innovation Silvercloud Health Ltd Dublin Ireland

**Keywords:** Web-based intervention, depression, adherence, engagement, eHealth, internet

## Abstract

**Background:**

Internet interventions can easily generate objective data about program usage. Increasingly, more studies explore the relationship between usage and outcomes, but they often report different metrics of use, and the findings are mixed. Thus, current evaluations fail to demonstrate which metrics should be considered and how these metrics are related to clinically meaningful change.

**Objective:**

This study aimed to explore the relationship between several usage metrics and outcomes of an internet-based intervention for depression.

**Methods:**

This is a secondary analysis of data from a randomized controlled trial that examined the efficacy of an internet-based cognitive behavioral therapy for depression (Space from Depression) in an adult community sample. All participants who enrolled in the intervention, regardless of meeting the inclusion criteria, were included in this study. Space from Depression is a 7-module supported intervention, delivered over a period of 8 weeks. Different usage metrics (ie, time spent, modules and activities completed, and percentage of program completion) were automatically collected by the platform, and composite variables from these (eg, activities per session) were computed. A breakdown of the usage metrics was obtained by weeks. For the analysis, the sample was divided into those who obtained a reliable change (RC)—and those who did not.

**Results:**

Data from 216 users who completed pre- and posttreatment outcomes were included in the analyses. A total of 89 participants obtained an RC, and 127 participants did not obtain an RC. Those in the RC group significantly spent more time, had more log-ins, used more tools, viewed a higher percentage of the program, and got more reviews from their supporter compared with those who did not obtain an RC. Differences between groups in usage were observed from the first week in advance across the different metrics, although they vanished over time. In the RC group, the usage was higher during the first 4 weeks, and then a significant decrease was observed. Our results showed that specific levels of platform usage, 7 hours total time spent, 15 sessions, 30 tools used, and 50% of program completion, were associated with RC.

**Conclusions:**

Overall, the results showed that those individuals who obtained an RC after the intervention had higher levels of exposure to the platform. The usage during the first half of the intervention was higher, and differences between groups were observed from the first week. This study also showed specific usage levels associated with outcomes that could be tested in controlled studies to inform the minimal usage to establish adherence. These results will help to better understand how to use internet-based interventions and what optimal level of engagement can most affect outcomes.

**Trial Registration:**

ISRCTN Registry ISRCTN03704676; http://www.isrctn.com/ISRCTN03704676

**International Registered Report Identifier (IRRID):**

RR2-10.1186/1471-244X-14-147

## Introduction

### Background

Internet- and computer-based interventions for depression have been shown to be effective in several meta-analytic reviews, reporting comparable effects to face-to-face treatments [1-5]. These findings have led to the use of these interventions within stepped care and collaborative care models of mental health provision, which operate on the premise that not everybody requires a high-intensity treatment in the form of face-to-face therapy provided by a trained psychologist [6]. The inclusion of these interventions into mental health services may lead to benefits, such as reduced costs associated with treatment, reduced waiting-list burden, and increased accessibility to services [7,8]; however, results are mixed, and more research is needed to draw firmer conclusions [9].

Despite the success of internet-based interventions, many questions about how these interventions work and for whom they are most suited remain unanswered. Most of the studies about internet-based cognitive behavioral therapy (iCBT) only analyze outcomes at fixed time points, treating the intervention as a singular entity, without considering the way the program is actually adopted and used by the users [10]. In this sense, some research has shown that the users’ uptake and long-term use of these technologies are lower than might be expected, and a median of 56% of the participants complete the whole program [11]. However, other research has shown that the users do not necessarily need to go through the entire program to benefit clinically [12]. Although treatment dropout and spontaneous remission might explain some of these trends, as they could in other psychological interventions, understanding more completely how internet-delivered interventions are used and whether their usage differs depending on user characteristics is an important area of investigation [13].

One of the benefits of internet-delivered interventions at a research level is that they facilitate the collection of objective data on usage and engagement. In recent years, several studies have turned their attention to the usage of internet-delivered intervention and how usage is related to outcomes [10,14]. Previous research has revealed a relationship between how much users were actually exposed to the program and outcomes; nevertheless, as different metrics of usage have been used across studies, and results are somewhat mixed, the specific contributions of these variables to outcomes remain unclear [15,16]. A review conducted by Donkin et al [15] compiled studies that looked at usage metrics and their relation to outcomes. Their findings showed that the number of log-ins and the number of modules completed were the most commonly reported metrics among different trials. Module completion was therefore found to be the most related metric to outcomes. However, these 2 metrics do not necessarily account for the depth of involvement of the users with the platform and its content. In this regard, it is possible to distinguish between *active* and *passive* engagement in internet-based interventions, where the former involves users interacting with the program and completing the activities, and the latter speaks about users who go through the program only superficially and therefore barely interacting with it [17,18]. Thus, to get the most from the intervention users’ exposure to the platform, one not only has to consider module completion but also completing prescribed activities and homework [19,20]. For this reason, exploring the relationship among the various available usage metrics through the development of composite metrics could shed more light on the actual effects of usage and adherence on outcomes [15]. In fact, Donkin et al [21] found that a composite measure (average number of activities completed per log-in) was the only predictor of clinically significant change, in contrast to other metrics, such as the time spent on the platform, the number of modules completed, or the number of log-ins. More studies are needed to explore composite metrics and their relation to outcomes, as this may inform the minimal dose of iCBT needed to achieve significant clinical benefit.

Furthermore, there is a lack of agreement in how adherence to treatment is actually defined and measured [16]. Adherence is often reported in terms of attrition or dropout from a trial [13], that is, the number of users who cease to use the intervention and therefore do not complete the per protocol treatment. However, several authors highlight that this information provides limited insight into users’ interaction with Web-based interventions and how their use might influence outcome measures [21]. On the other hand, recent studies have conceptualized adherence as the intended use of the platform or “the extent to which individuals should experience the content to derive maximum benefit as implied by its creators” [22], which is also known as the therapeutic dose [16]. Following Sieverink’s argument [16], intended use should be considered the minimum use to establish adherence; however, current electronic health evaluations fail to demonstrate the optimal dose-response relationship. In this sense, some authors suggest that designers need to find the balance between the theoretically efficacious dose and the effective or actual dose that can be only determined upon application [23]. Therefore, finding the most relevant metrics and determining thresholds of usage for these metrics are key to determining the optimal dosage, which in turn may be different depending on the target population, setting, or the type of intervention that is evaluated [23].

Another important factor that can have an impact on the efficacy of and adherence to internet-based interventions is the role of support [24]. Support may take different forms, but generally speaking, it involves someone checking in with the patient, encouraging the user to continue going through the platform, and providing feedback on the basis of the patient’s progress. Different meta-analytic reviews have shown that supported interventions have higher rates of adherence and better outcomes than self-guided ones [1,3,4,25]. However, the amount and frequency of the support needed to produce clinically significant improvements are barely understood and results so far do not show differences among different doses of support [24,26,27]. In this sense, no studies have explored the role of metrics related to the support, such as the number of reviews and the number of user replies to these reviews, which could shed more light on the role of support in the usage of the program and their outcomes [28].

In summary, the literature shows that there is an association between higher usage leading to better outcomes, but it remains unclear which of these usage metrics are more strongly related to outcomes. Furthermore, few studies have attempted to determine an optimal dose-response relationship that could inform a threshold to establish adherence.

### Objectives

This study was aimed at exploring the relationship between several usage metrics and outcomes from a sample of individuals with depressive symptoms and who were involved in a trial that evaluated the efficacy of a Web-based supported intervention for depression [29,30]. The specific goals of the study were as follows: (1) to explore the differences in usage between those who significantly improved and those who did not, (2) to analyze differences in usage across different sociodemographic and clinical variables of both groups, (3) to explore which of the usage metrics were more important in predicting clinically significant changes, and (4) to explore whether specific usage levels are associated with a clinically meaningful change.

## Methods

### Study Design

This study is a secondary analysis of data from a randomized controlled trial (RCT) that examined the efficacy of an iCBT intervention for depression in a sample of adults from a community setting. The protocol and the main outcome paper have been published elsewhere [29,30]. In the main study, participants were randomized to the internet-delivered intervention with support or the waiting-list control group. Assessment took place at baseline and at posttreatment, 8 weeks after randomization. The study protocol, information on the study, informed consent, and related materials were approved by the ethics committee at the School of Psychology, Trinity College Dublin (November 22, 2013). Participants who were excluded from the main RCT also completed consent, agreeing to have their data included for analysis. The trial is registered as a controlled trial with ISRCTN (ISRCTN03704676).

### Sample and Recruitment

For details on the participant flow and characteristics, see Richards et al [29]. In summary, 641 users from the Aware charity expressed interest and applied to participate in the research. From them, 379 users were excluded for different reasons, such as Beck Depression Inventory 2nd edition (BDI-II) scores <14 (n=114); BDI-II>28 (n=211); suicidal intent/ideation (n=16); if they were currently receiving psychological treatment for depression (n=104); organic mental health condition (n=82); on medication for less than 1 month (n=106); alcohol or drug misuse (n=50); and reported depressive symptoms that preceded or coincided with a diagnosed medical condition (n=138). Even though these excluded participants were not included in the trial, they were offered the intervention with support, and they were also administered the primary outcome measures. The only difference between those individuals who were included and excluded from the trial is that the latter were not actively followed up to complete the posttreatment measures. For the purposes of this study and given that those participants excluded in the trial received the same intervention, all participants who logged in to the platform and completed the outcome measures upon completion of their treatment were included in the secondary analyses. Similarly, all those participants who were assigned to the waiting-list group and received the intervention after the waiting-list period were included in the secondary analyses, taking as their posttreatment scores the scores they provided upon completion of the intervention. With regard to those participants who were not part of the main RCT, we only selected those who completed the posttreatment outcomes within a period of 85 days after the first log-in. This time period was computed by calculating the average number of days that participants included in the trial took to fill the posttreatment measures (mean 66.86, SD 9.15), and 2 SDs were added. Thus, we excluded users who completed posttreatment measures beyond the intended assessment period.

### Procedure

The study was advertised through the Aware website, and those individuals who expressed an interest to participate were emailed about the intervention study and directed to a website to access further information on the study and what would be involved in participating. Informed consent and baseline screening questionnaires were completed on the Web. Thereafter, those participants who met the inclusion criteria were randomly assigned to the intervention group or the waiting-list group, and those who did not meet the inclusion criteria but were interested in participating were also given access to the intervention. Once a participant was assigned to the active treatment at the first log-in, the participant received a message from the participant’s supporter, and this support was then offered once a week for a period of 8 weeks. At the end of the 8-week period, participants were automatically asked to complete the outcome measures, and those in the intervention group of the trial, who did not complete the measures, were followed up by the research team to achieve the completion.

### Intervention

#### Computerized Cognitive Behavioral Therapy Program

Space from Depression is a 7-module Web-based, cognitive behavioral therapy–based program for depression. This program was developed by SilverCloud Health, which is a company that develops Web-based interventions for mental health conditions. The intervention is delivered on a Web 2.0 platform, using media-rich interactive content. The treatment comprises cognitive and behavioral components, including self-monitoring and thought recording, behavioral activation, cognitive restructuring, and challenging core beliefs. These components are included across the 7 modules, although the program follows a nonlinear fashion, which means that the user can go directly to the module that is of interest to him or her. Each module follows a structured format that includes introductory quizzes, audios, videos, informational content, personal stories, interactive activities, and homework suggestions. Space from Depression has been described in detail elsewhere [29,30].

#### Support

Participants were assigned a trained supporter who monitored their progress throughout the trial. These supporters were trained volunteers of the charity who received training in the SilverCloud platform and on how to deliver feedback. A dashboard interface provided supporters with an overview of their participants’ level of engagement with the program content. The role of the supporter mainly comprised encouraging, supporting, and providing feedback to the users, and this feedback used to take between 10 and 15 min per participant. Support was offered once a week during the period of 8 weeks.

### Measures

#### Primary Outcome

The primary outcome of the main RCT was the BDI-II [31]. The 21-item measure is a widely used questionnaire that assesses severity of depressive symptoms using a Likert scale ranging from 0 to 3 on the basis of the Diagnostic and Statistical Manual of Mental Disorders, 4th edition, diagnostic criteria. The scale designates levels of severity, minimal (0-13), mild (14-19), moderate (20-28), and severe (29-63) [31]. This instrument has shown good psychometric properties in several studies.

#### Usage Metrics

##### Total Time on the Platform

This metric corresponds to the combination of the time spent in each session (in min) from the first to the last log-in. Interactions lasting longer than 30 min are automatically counted as 1 min, to avoid counting long idle periods when the program is open toward the total count.

##### Number of Sessions

This metrics relates to the number of times (log-ins) the user accessed the program. If a specific session has inactivity periods longer than 3 hours, the next moment of activity will count as a new session.

##### Average Time (in min) Per Session

This composite measure results from dividing the total time on the platform by the number of sessions.

##### Number of Activities

This metric is calculated by counting all the times users interacted actively with the platform, that is, every time that they completed a journal entry, used an interactive tool, or downloaded or played relaxation audios. The program has a total of 17 interactive activities distributed across the 8 modules. Participants were able to use these activities as many times as they wished.

##### Activities Per Session

This is a composite measure resulting from dividing the number of activities completed by the number of sessions.

##### Percentage of the Program Viewed

This metric refers to the percentage of the total program content that the user has gone through.

##### Number of Reviews

This metric refers to the number of messages that the supporter sent to the user to encourage use of the platform while providing feedback about the progress from the last review.

##### Number of Review Notes

This metric relates to the number of replies that the user left for their supporter after a review.

### Data Analysis

Data analyses were completed using SPSS 24 (IBM corporation). In the first place, *t* tests and chi-square tests were computed to explore potential differences in sociodemographic and clinical variables at baseline between those participants who met the inclusion criteria for the trial and those who did not. Boxplots of the sample were computed to look for any extreme outliers (3 box lengths away from the edge of their box). One-way analysis of variance (ANOVA) analyses and *t* tests were conducted to explore differences in usage across different sociodemographic and clinical variables at baseline. Pairwise comparisons applying Bonferroni correction were conducted between the subgroups associated with each category. An assessment of reliable change (RC) was made using criteria of a change of ≥9 points or greater on pre-to-post treatment BDI-II scores. Similar criteria have been used in other studies of internet-delivered interventions for depression [19,32,33]. *t* tests were performed to explore differences in the usage metrics between those who obtained an RC and those who did not. Variables identified as significantly associated with RC were further examined; 2×8 repeated-measures ANOVA were conducted to explore the change in the scores across the 8-week intervention period, comparing those who obtained an RC and those who did not. When sphericity was violated, Greenhouse-Geisser correction was applied for ANOVA analyses. Pairwise comparisons applying Bonferroni correction for multiple comparisons were conducted within groups, comparing week 1 with further weeks, and between groups, comparing the outcomes between groups for each specific week. To obtain the optimal cutoff for achieving RC, a receiver operating characteristic (ROC) curve analysis [34] was performed using each of the 4 individual usage metrics (total time spent on the platform, number of sessions, number of activities, and percentage of the program viewed) as test variables against the *RC* state variable. ROC curves are constructed by plotting true positive rates (sensitivity) against the false positive rates (specificity). The optimal cutoff was determined using the *point of curve closest to the (0,1)* criteria, which uses the formula *d2=[(1–Sn)2* *+(1–Sp)2]*, where Sn = sensitivity and Sp = specificity, to calculate the distance of each point to the (0,1) point representing maximal sensitivity and specificity [35].

## Results

### Overview

**Figure 1:**
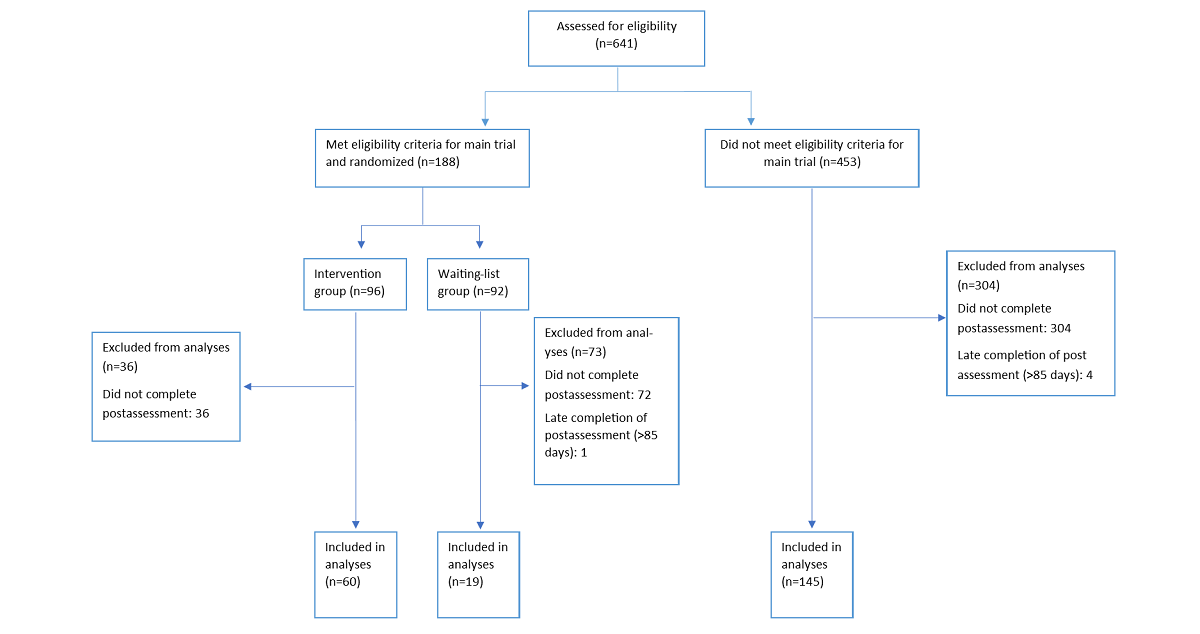
Flowchart of the current study.

Of the 641 participants who showed interest in the study and provided consent, a total of 224 participants provided postintervention outcome data within a period of 85 days. Figure 1 illustrates the number of participants coming from each of the branches of the main trial. The number of participants included in this study that were excluded from the main RCT, and the reasons are as follows (participants can respond to more than one reason):

- BDI-II score<14 (n=52)
- BDI-II score>28 (n=56)
- Suicidal intent/ideation (n=3)
- Currently receiving psychological treatment for depression (n=30)
- Organic mental health condition (n=14)
- On medication for less than 1 month (n=26)
- Alcohol or drug misuse (n=14)
- Age<18 (n=1)
- Depressive symptoms that preceded or coincided with a diagnosed medical condition (n=46)

To ensure the homogeneity of the sample in terms of sociodemographic measures and usage metrics between those participants who met the inclusion criteria for the RCT (treatment group and waiting list) and those who were excluded, baseline differences in clinical and sociodemographic measures and usage metrics were examined. There were no differences between these groups in age, gender, marital status, BDI-II pre and postintervention, or any of the usage metrics included in the study. Before further statistical analyses, box plots were constructed for the different usage metrics to spot extreme outliers (3 box lengths away from the edge of their box). Owing to the high variability of the values on the usage metrics and to prevent the distribution of the sample to be very skewed, 8 participants were classified as extreme outliers, and they were excluded from further analyses, leaving a total sample of 216. To illustrate the type of outlier, one example was that of an individual user who spent a total time of 2526 min (42.11 hours) and completed 117 sessions and 321 activities, which is unrealistically far away from the average found in this study. In other words, these participants deviated substantially from the different usage patterns that an individual might take in this specific program.

### Platform Usage

Descriptive analyses of the total usage of the platform show that, on average, participants spent 339 min on the platform, they accessed the program 14 times, viewed 57% of the total program, completed 25 activities, got 7.5 reviews from the supporters, and left 1.62 messages to their supporters. ANOVA analyses were conducted to explore potential differences in the total usage of the platform across sociodemographic and clinical variables (Multimedia Appendix 1). Regarding age groups, univariate ANOVA models showed significant differences in the number of activities completed (*F*_3,212_=4.03; *P*=.008) and activities per session by age group (*F*_3,212_=2.95; *P*=.03). Pairwise comparisons showed that individuals in the age group of 31 to 40 years completed significantly more activities (mean difference=15.06, SE 5.65; *P*=.049) and more activities per session (mean difference=0.95, SE 0.34; *P*=.03) than those older than 50 years. Regarding depressive symptom severity at baseline, univariate ANOVA models showed significant differences between depression severity groups at baseline for total time spent (*F*_3,212_=2.65; *P*=.049), number of sessions (*F*_3,212_=5.75; *P*=.001), number of activities completed (*F*_3,212_=2.87; *P*=.04), percentage viewed (*F*_3,212_=2.82; *P*=.04), and number of reviews (*F*_3,212_=3.7; *P*=.01). Pairwise comparisons showed that users with minimal depressive symptoms had lower engagement overall. This group had significantly lower usage rates as compared with those with severe symptoms in terms of number of sessions (mean difference=6.31, SE 1.74; *P*=.002), number of activities (mean difference=14.19, SE 5.08; *P*=.03), and number of reviews (mean difference=0.78, SE=0.25; *P*=.01). It also had significantly lower number of sessions (mean difference=5.56, SE 1.66; *P*=.006) and percentage of program viewed (mean difference=0.15, SE 0.05; *P*=.04) as those with moderate depression, and it had significantly lower number of sessions than those with mild depression at baseline (mean difference= 6.12, SE 1.9; *P*=.009).

### Platform Usage Associated With Reliable Change in Beck Depression Inventory 2nd Edition Scores

The sample of participants included in the study was divided between those who obtained an RC in the BDI-II (reduction of 9 points or greater on pre-to-post treatment BDI-II scores) and those who did not. Overall, 89 participants (41%, 89/216) reached an RC, and 127 (59%, 127/216) did not obtain an RC. *t* tests were run to determine whether there were differences in the usage of the platform between users of both groups. Results (see Table 1) showed significant differences in the total time spent on the platform (time spent), number of sessions, program viewed (percentage viewed), total activities completed (number of activities), and number of reviews in favor of those who obtained an RC, showing medium between-group effect sizes (Cohen *d*=0.45-0.61). No significant differences were obtained for min per session, activities per log-in, and number of review notes.

**Table 1:** Descriptive data and mean differences in usage metrics between those who reliably changed and those who did not.

Usage metrics	Reliable change (n=89), mean (SD)	No reliable change (n=127), mean (SD)	*t* test (df)	*P* value	Effect size (Cohen *d*)
Time spent	420.63 (280.77)	282.23 (253.24)	3.78 (214)	<.001	0.52
Sessions	17.63 (8.93)	12.22 (8.92)	4.38 (214)	<.001	0.61
Percentage viewed	67.56 (25.24)	50.46 (30.41)	4.50 (207.87)	<.001	0.61
Activities	33.28 (29.42)	19.62 (22.97)	3.67 (159.01)	<.001	0.52
Reviews	7.83 (0.77)	7.28 (1.57)	3.39 (194.60)	.001	0.45
Reviews notes	1.94 (2.29)	1.39 (2.10)	1.85 (214)	.06	0.25
Min per session	24.46 (13.43)	22.68 (16.86)	0.83 (214)	.41	0.12
Activities per session	1.84 (1.35)	1.53 (1.74)	1.41 (214)	.16	0.20

^a^Bonferroni correction applied (alpha=.05/8=.006).

### Weekly Usage of the Platform

**Figure 2:**
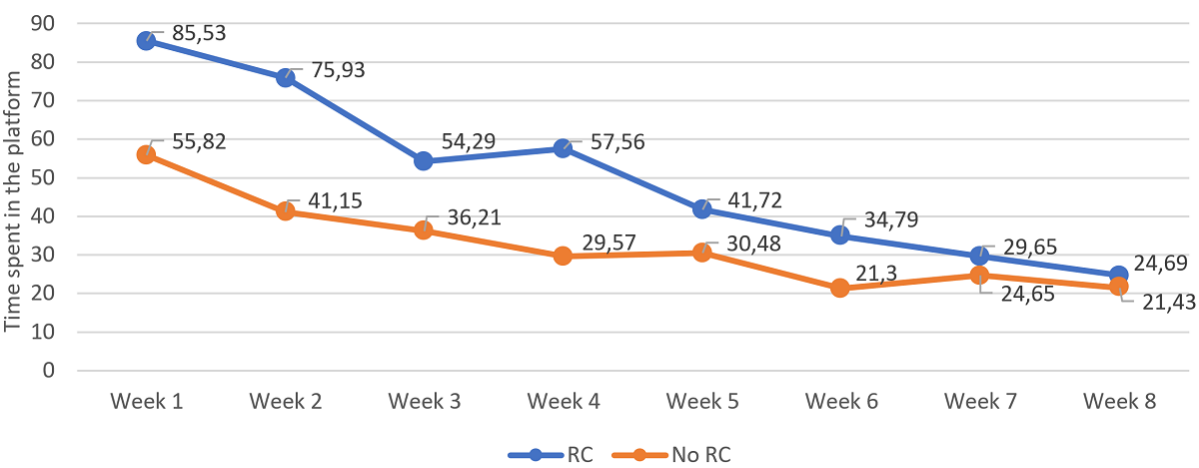
Time spent on the platform per week and divided between those who got a reliable change (reduction of 9 points or greater on pre-to-post treatment Beck Depression Inventory 2nd Edition) and those who did not. Significant differences were found in between-group comparisons at weeks 1-4 and week 6; significant within-group differences were found between week 1 and weeks 5-8 for the reliable change (RC) group; significant within-group differences were found between week 1 and weeks 6-8 for the no RC group.

**Figure 3:**
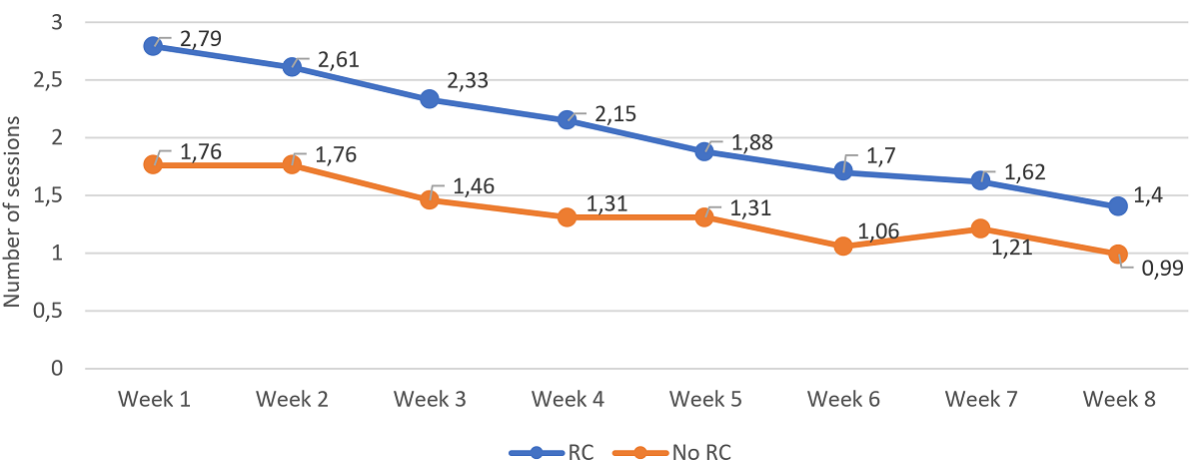
Number of sessions performed per week and divided between those who got a reliable change (reduction of 9 points or greater on pre-to-post treatment Beck Depression Inventory 2nd Edition) and those who did not. Significant differences were found in between-group comparisons at weeks 1-8; significant within-group differences were found between week 1 and weeks 5-8 for the reliable change (RC) group; significant within-group differences were found between week 1 and weeks 6 and 8 for the no RC group.

**Figure 4:**
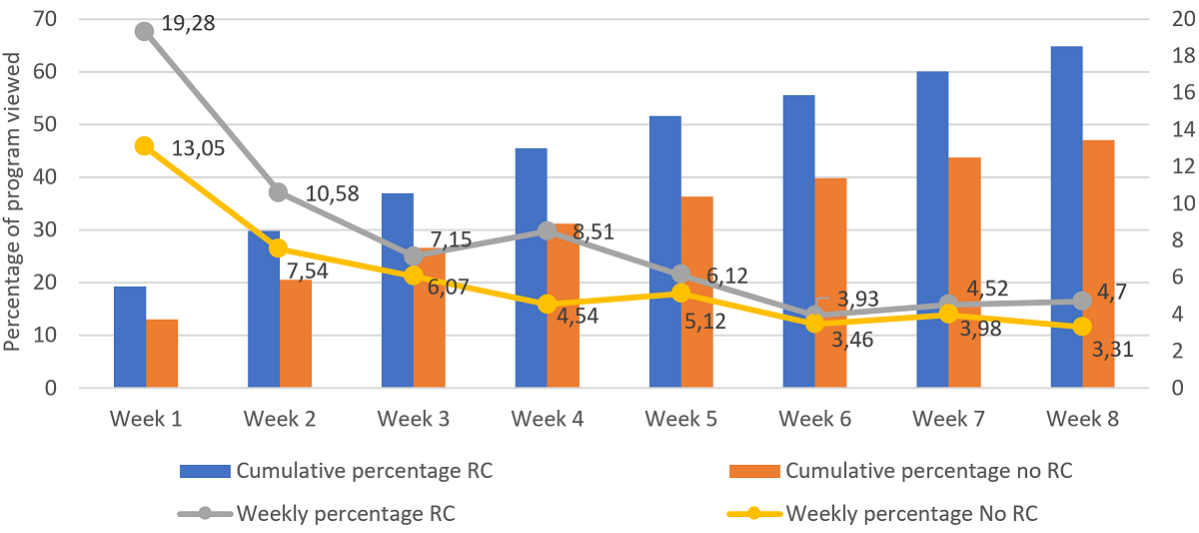
Percentage of the program viewed per week and divided between those who got a reliable change (reduction of 9 points or greater on pre-to-post treatment Beck Depression Inventory 2nd Edition) and those who did not. Significant differences were found in between-group comparisons at weeks 1, 2 and 4; significant within-group differences were found between week 1 and weeks 2-8 for the reliable change (RC) group; significant within-group differences were found between week 1 and weeks 2-8 for the no RC group.

**Figure 5:**
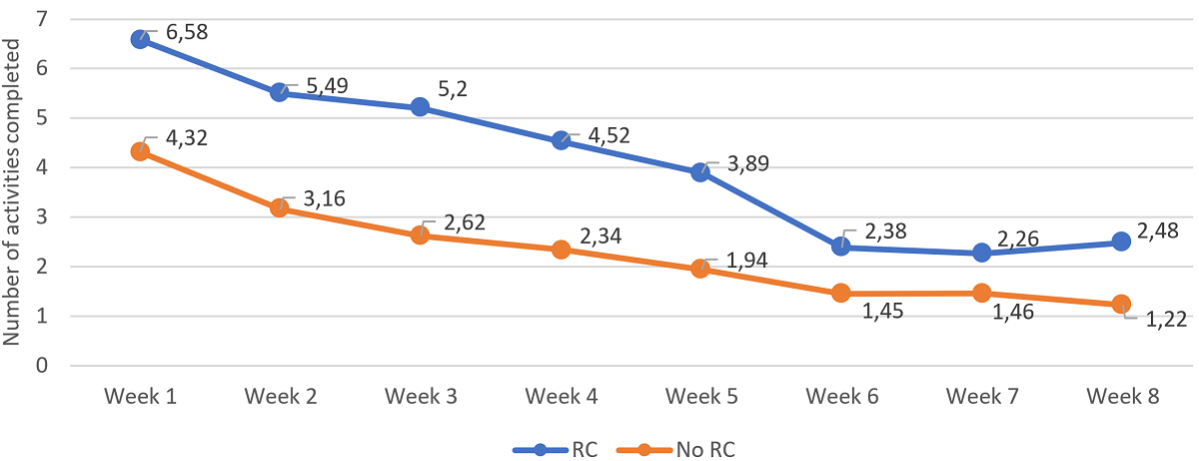
Number of activities completed per week and divided between those who got a reliable change (reduction of 9 points or greater on pre-to-post treatment Beck Depression Inventory 2nd Edition) and those who did not. Significant differences were found in between-group comparisons at weeks 1-6 and 8; significant within-group differences were found between week 1 and weeks 5-8 for the reliable change (RC) group; significant within-group differences were found between week 1 and weeks 6 and 8 for the no RC group.

Weekly usage of the program was explored among the 4 usage variables that were significant in the previous analyses, namely time spent, number of sessions, number of activities, and percentage viewed (Figures 2-5). Number of reviews were not included, as all participants got 1 review per week, as this was established in the protocol, unless they dropped out from the treatment. To explore potential differences in these variables over time and between individuals who showed RC and individuals who did not among the 8-week intervention period, 2×8 repeated-measures ANOVA analyses were computed. With regard to the time spent, analyses showed significant time (*F*_4.58,980.08_=21.91; *P*<.001) and interaction effects (*F*_4.58,980.08_=2.77; *P*=.02). Pairwise comparisons between groups showed significant differences between groups in the time spent on the platform from week 1 to 4 and week 6. Pairwise comparisons within groups showed that, in the group of individuals with RC, the time spent in week 1 was not significantly different compared with weeks 2, 3, and 4, but it was significantly higher compared with week 5 and the following weeks, indicating that the time spent during the first 4 weeks was longer than the following 4 weeks.

Regarding the number of sessions, there was a significant difference in time (*F*_4.99,1067.45_=21.03; *P*<.001); however, interaction effects were not significant (*F*_4.99,1067.45_=1.82; *P*=.11), indicating that, altogether, there were no differences between conditions in the number of sessions performed. However, pairwise comparisons showed significant differences between groups in the number of sessions in each week across the 8-week period. Pairwise comparisons within the RC group showed nonsignificant differences in the number of sessions done in week 1 compared with weeks 2, 3, and 4. However, when comparing week 1 to 5 and following weeks, significant differences were observed, indicating again that the number of sessions was more similar from week 1 to 4, and there was a significant decrease in the number of sessions in the following weeks.

With regard to the percentage viewed, ANOVA analysis showed significant time (*F*_4.73,1012.57_=43.96; *P*<.001) and interaction effects (*F*_4.73,1012.57_=2.71; *P*=.02). Between-group pairwise comparisons showed significant differences in the percentage viewed in weeks 1, 2, and 4, and this percentage was nonsignificant for the other weeks. Pairwise comparisons within the RC group showed that the weekly percentage viewed in the first week was significantly higher compared with all the other weeks.

Finally, regarding the number of activities, ANOVA analysis showed significant effects for time (*F*_5.12,1095.63_=16.64; *P*<.001), but it did not show significant effects for the interaction between groups (*F*_5.12,1095.63_=1.12; *P*=.35), indicating that there were no overall differences between conditions in the number of activities completed. Between-group pairwise comparisons showed significant differences in the number of activities completed in nearly all the weeks, with the exception of week 7. Within-group pairwise comparisons of the group with RC showed that there were significant differences only when comparing the number of tools used between week 1 and week 5 and the following weeks.

Overall, these analyses show that the weekly usage was consistently larger for the group with RC in the different metrics among the first half of the intervention period, and from here, the differences between groups started to reduce. Within the RC group, a pattern can be observed across nearly all of the metrics where the usage of the first week was not significantly different from the usage on weeks 2, 3, and 4, but it was significantly different from week 5 in advance.

### Receiver Operating Characteristic Curve Analysis

A total of 4 separate ROC curve analyses were run with the following results: for total time spent on platform, the optimal cutoff was 420 min, with a specificity of 76% and sensitivity of 47%. For number of sessions, the optimal cutoff was 15, with a specificity of 71% and a sensitivity of 61%. For the percentage of the program viewed, the optimal cut-off was 50%, and this was related to a specificity of 54% and a sensitivity of 79%. Finally, for number of activities, the optimal cut-off was 30 tools used, which had a specificity of 77% and a sensitivity of 46%. The area under the curve values ranged from .65 to .68, with 95% CIs between .58 and .76, considered within an acceptable range for using these variables to differentiate between those who reliably changed and those who did not show RC as defined in the study as a movement of ≥9 points on the BDI-II. When using these cutoffs as measures of intended use, those using the platform for at least 420 min or 7 hours (n=62) had a 58% RC rate, those logging in for at least 15 sessions (n=91) had a 59% RC rate, those using tools (activities) at least 30 times (n=61) had a 57% RC rate, and those using at least 50% of the program (n=115) had a 52% RC rate. All these values are significantly higher than the overall RC rate of 41% for the entire sample. Finally, of those who reached all 4 optimal cutoffs (n=34), the RC rate was 62%.

## Discussion

### Principal Findings

This study was intended to explore the relationship between usage of the platform and the outcomes in a sample of participants who used an iCBT intervention for depression. Overall, the results showed that those individuals who obtained an RC after the intervention had higher levels of exposure to the platform, in terms of time spent, number of sessions, percentage of the program viewed, and number of activities, compared with those who did not. Differences in program usage between those who improved and those who did not were observed from the first week, although these differences started to vanish during weeks 5 to 8 of the intervention. Furthermore, in the RC group, the usage of the platform during the first week was not significantly different in comparison to the first half of the intervention period (until week 4), and these differences turned significant when compared with week 5 in advance, indicating a significant reduction of the usage during the second half of the intervention period. Finally, independent ROC curve analyses showed that 7 hours of time spent on the platform, logging in 15 times, and completing 30 activities over the intervention course were associated with the achievement of RC. In other words, the likelihood of being in the RC group, compared with the likelihood of being in the no RC group, was highest at these thresholds.

With regard to the sociodemographic and clinical variables and their relation to program usage, our results showed that individuals older than 50 years completed fewer activities compared with younger cohorts. These results add up to the contradictory literature about age and adherence, where different studies are finding different directions in the relationship between age and adherence [36,37]. It may be the case that age on its own is not as important as the interaction of age with other factors, such as computer literacy, which makes a difference in this relationship. This study also showed that individuals with minimal depressive symptoms at baseline had significantly lower levels of usage compared with users with higher levels of symptomatology. This could be explained by the fact that the intervention is ideally intended for individuals with mild-to-severe depression, and this cohort of less depressed users did not require the same grade of exposure to get benefits, or the program indeed fell outside their needs, as it is a treatment intervention. In this sense, these participants might benefit more from preventive approaches, such as a resilience intervention. Our results here highlight the necessity and importance of delivering the most appropriate intervention at the right time. Still, it is worth mentioning that although the literature in this regard shows mixed results [36], a related study identified important benefits of the Space from Depression program for those with subclinical symptoms [38].

In this study, on average, the users utilized 57% of the program, which is similar to completion rates in other internet-based interventions for depression [36,39]. When comparing the usage between those who obtained an RC and those who did not, results showed that the former significantly spent more minutes, accessed more times, completed more activities, were exposed to more content, and received more reviews. It should be noted that the time spent on the platform by those who achieved an RC was around 7 hours, which is slightly higher but still similar to the 6 hours found in previous studies, and the time spent by those who did not achieve an RC is similar with previous results reporting between 4 and 5 hours [21,37]. However, the literature around the relationship between usage and outcomes in internet-based interventions for mental health shows contradictory results, as variables, such as time spent and number of sessions, are not consistently related to outcomes, and studies with greater statistical power need to be conducted to shed more light on this relationship [15]. Our results confirm the largely used statement *the more usage, the better* [5,16], although it is important to note that our variables account for *active* (ie, activities completed) and *passive* (ie, percentage viewed) engagement, and both elements are important to get the most out of these interventions [20]. On the other hand, composite measures, such as minutes per session and activities per session, were not significantly different between conditions, which contradicts results obtained by Donkin et al [21]. The nonlinear fashion of Space from Depression and the fact that this program is very focused on the usage of tools might explain the absence of differences between these groups in their behavior within sessions, where the amount of time and tools used was not significantly different between those who achieved an RC and those who did not.

With regard to the weekly usage, our results showed that participants who obtained an RC had significantly larger exposure levels to the intervention compared with those who did not improve, and these differences were more consistent among the first 4 weeks. Focusing on the usage over time of those who obtained an RC, the results showed similar usage levels among the first 4 weeks, but the results showed a significant subsequent decrease in the second half of the intervention. Overall, these results are in line with another study, where the program was mostly used during the first half of the intervention period [37]. In this sense, these results suggest that the usage levels during the first month might be key for improvement, and strategies for enhancing engagement at this stage could be beneficial. One of these strategies could be outcome and engagement monitoring, inbuilt in the feedback system, so that participants could be flagged if they were deviating from the expected results of the intervention, and causes for this could be explored and addressed [40]. In a similar vein, a recent study found that when therapists were given outcome feedback about patients who were deteriorating during the intervention period, these patients had significantly less severe symptoms after treatment compared with similar patients assigned to therapists who were not receiving this feedback [41].

The ROC curve analyses are exploratory in nature, and the results do not allow to draw firmer conclusions about optimal usage levels; however, they can be understood as a first step toward determining specific thresholds that could be tested in controlled and experimental designs. As recommended by some authors, the optimal dose needs to consider the balance between user’s burden and adherence to ensure that the effective (observed) dose is as close as possible to the efficacious dose, which is not dependent on adherence [23]. For this specific intervention, the maximal efficacious dose is to complete the 7 modules during an 8-week period at the pace of 1 module per week; however, our findings seem to indicate that the effective dose would not require the completion of all the modules. In 3 out of 4 measured variables, the specificity of the optimal dose was high, and the RC rates were close to 60% in those who reached this usage cutoff. The optimal cutoff for the percentage of program viewed variable showed low specificity, signifying that a minimal 50% use of the platform would have a high percentage of false positives, not ideal for determining intended use. Overall, in this population at least, 7 hours of platform usage spread out over 15 sessions and completion of 30 activities (these include repeat activities for learning key skills) over a maximum period of 12 weeks were associated with achieving a clinically significant change. Nevertheless, these results have to be considered within their context, and further studies with changes to settings, types of programs used, and other study features, such as population characteristics, may yield different results. However, the parameters found for this particular intervention are worth exploring further.

This paper contributes to the concept of adherence by providing an empirical justification of intended use, that is, “the extent to which individuals should experience the content to derive maximum benefit from the intervention, as defined or implied by its creators” [22]. As suggested by different authors, there is a need for demonstrating the dose-response relationship, and this paper has done so through the consideration of different metrics. In this sense, future studies should explore whether similar exposure levels are needed for RC in different platforms, and future studies should determine which are the actual tools that have been used as some studies have already done [37]. Although our results will help identify optimal levels of exposure to maximize the benefits from these interventions, future studies should be conducted to go deeper into the usage patterns, as they can take many different pathways, and different types of usage can lead to benefits [42]. Log data of those participants who reliably improved would be a way of understanding different successful patterns of usage and which tools or modules are most related to change.

### Limitations

This study also has some limitations. First, although the sample of this study comes from an RCT, this substudy was observational in nature, and no manipulation of the variables related to usage was done. Given this, it is not possible to establish causal relationships between usage metrics and outcomes. Future analyses, such as cross-lagged models, could focus on whether higher usage rates lead to lower symptoms over time or whether decreasing symptoms is what drives higher usage. This may answer the question of causality in this association. RCTs could also look into comparing whether a group pushed and receiving recommendation for reaching certain usage levels will perform better than a group permitted to use an intervention freely. Moreover, the inclusion of only those participants who reported posttreatment outcomes might be limiting the generalizability of the results, as it has been found that participants who engage more are also more likely to respond to follow-up assessments [43]. Thus, it might be possible that levels of usage were lower for those who did not complete the postassessments. Another limitation relates to the number of reviews variable, as supporters were encouraged to offer reviews regardless of whether participants were actively using the platform. Future studies should provide different indications to the supporters, so that the supporters do not need to waste time writing reviews for users who are not logging in to the platform. For example, supporters could have 2 attempts to contact participants who are not using the platform and then discharge them from services if the answer is not given. Finally, the metric percentage viewed, which only accounts for new content viewed, not considering content reviewed, has been shown as a key element of internet-based interventions usage [39]. These results could also explain why the relationship of this variable with outcomes is not as clear as the others.

### Conclusions

This study has used different ways to explore the relationship between usage of an iCBT intervention for depression and the outcomes achieved by participants. The results seem to reinforce the notion that *the more usage of a Web-based intervention, the better* but with some nuances. Thus, the usage during the first half of the intervention was significantly higher compared with the second half, which might have implications for engagement and how best to offer the support in the early stages. Furthermore, this study suggests that it may be possible to determine, at least preliminarily, an optimal dose that would need to be tested and replicated to draw firmer conclusions. If confirmed, these thresholds could be used to establish cutoffs of adherence to the intervention. Future studies should continue to explore the relationship between usage and outcomes to better understand how internet-delivered interventions work and how to make them more responsive to varying degrees of usage. The continuation of this line of research could lead us to a future where a responsive intervention takes into account usage levels, allowing for tailoring in real time to enhance the engagement of different participants and thus maximizing likelihood of a positive outcome.

## Appendix

Multimedia Appendix 1 Mean and SDs of usage metrics of those who completed posttreatment outcomes (n=216) divided by clinical and sociodemographic factors.

## Abbreviations

ANOVA: analysis of variance
BDI-II: Beck Depression Inventory 2nd edition
iCBT: internet-based cognitive behavioral therapy
RC: reliable change
RCT: randomized controlled trial
ROC: receiver operating characteristic
