# Supplementary material for: Exploring the Relationship Between Usage and Outcomes of an Internet-Based Intervention for Individuals With Depressive Symptoms: Secondary Analysis of Data From a Randomized Controlled Trial
Source: J Med Internet Res. 2019 Aug 1;21(8):e12775. doi: 10.2196/12775 (PMC6694731; doi:10.2196/12775)
Supplement: Multimedia Appendix 1 [file jmir_v21i8e12775_app1.pdf]

Supplementary Table 1. Mean and standard deviations of usage metrics of those who completed post-treatment outcomes (n=216) divided by clinical and sociodemographic factors

	Total Time spent (min)	Number of sessions	Average time per session	Number of activities per session	Percentage viewed	Activities completed	Number of reviews	Number of review notes
**Age group**								
Up to 30	339.04 (283.17)	14.91 (10.78)	21.30 (11.37)	1.83 (1.33)[a]	60.67 (30.17)	28.78 (28.07)[a]	7.7 (1.04)	1.68 (2.28)
31-40	340.89 (229.67)	14.59 (8.07)	24.46 (16.06)	2.02 (2.04)[a]	61.07 (29.51)	29.13 (28.32)[a]	7.58 (1.34)	1.55 (1.98)
41-50	363.91 (308.83)	14.89 (8.74)	21.83 (11.46)	1.29 (1.17)[a]	54.02 (27.31)	22.21 (23.98)[a]	7.53 (1.12)	1.79 (2.35)
>50	291.95 (296.06)	12.43 (10.36)	27.44 (24.45)	1.07 (1.11)[a]	48.60 (31.22)	14.07 (20.40)[a]	6.93 (1.91)	1.37 (2.33)
**Gender**								
Male	384.54 (320.80)	14.58 (9.23)	26.96 (18.82)	1.53 (1.87)	54.00 (29.33)	21.75 (24.43)	7.25 (1.33)	1.51 (2.02)
Female	324.54 (254.90)	14.45 (9.30)	22.26 (14.17)	1.70 (1.50)	59.00 (29.56)	26.38 (27.28)	7.60 (1.32)	1.65 (2.25)
**Education**								
High school/ other	347.84 (264.84)	14.86 (8.10)	22.72 (13.41)	1.45 (1.17)	57.17 (29.52)	23.62 (23.35)	7.53 (1.11)	1.63 (2.51)
Undergraduate	316.41 (252.88)	14.06 (9.08)	22.16 (13.90)	1.63 (1.57)	57.02 (28.03)	23.97 (24.09)	7.51 (1.70)	1.52 (1.98)
Postgraduate	353.80 (313.01)	14.23 (11.46)	26.28 (20.29)	2.08 (2.13)	58.74 (32.02)	29.81 (34.20)	7.47 (1.14)	1.72 (1.84)
**Employment**								
Unemployed	371. 77 (302.47)	14.72 (9.19)	26.69 (19.58)	1.67 (1.51)	57.54 (31.51)	25.53 (27.37)	7.40 (1.39)	1.90 (2.72)
Part time	326.42 (244.05)	15.19 (9.84)	20.57 (10.51)	1.57 (1.33)	56.65 (28.23)	26.71 (29.42)	7.61 (1.26)	1.46 (1.74)
Full time	326.96 (271.57)	13. 84 (9.07)	23.11 (15.03)	1.66 (1.59)	58.00 (29.41)	24.19 (24.53)	7.51 (1.33)	1.54 (2.08)
**Civil status**								
Single	325.65 (247.00)	14.30 (9.81)	23.81 (14.98)	1.84 (1.74)	58.47 (29.43)	25.27 (25.41)	7.58 (1.12)	1.51 (1.99)
Married	349.08 (290.61)	14.56 (9.00)	23.04 (15.20)	1.55 (1.51)	57.04 (29.86)	25.56 (28.15)	7.45 (1.48)	1.69 (2.37)
Divorced/ widowed	319.76 (245.92)	14.21 (9.96)	24.74 (21.60)	1.79 (1.57)	56.77 (29.46)	22.29 (18.38)	7.71 (0.73)	1.50 (1.45)
**Dependents**								
No	339.20 (281.75)	13.97 (9.58)	23.72 (14.56)	1.89 (1.74)[a]	59.22 (29.93)	27.53 (28.68)	7.53 (1.32)	1.61 (2.28)
Yes	339.34 (263.65)	15.02 (8.97)	23.05 (16.67)	1.40 (1.37)[a]	55.48 (29.14)	22.56 (23.83)	7.48 (1.35)	1.63 (2.09)

**Currently on medication**								
No	334.24 (271.28)	14.09 (9.31)	23.74 (15.97)	1.61 (1.45)	57.16 (29.31)	24.82 (26.90)	7.52 (1.26)	1.57 (2.11)
Yes	351. 45 (278.84)	15.33 (9.28)	22.60 (14.48)	1.78 (1.91)	58.35 (30.38)	26.29 (26.13)	7.48 (1.50)	1.73 (2.38)
**Currently on counselling**								
No	333.03 (274.39)	14.18 (9.35)	23.37 (15.52)	1.64 (1.60)	56.01 (29.64)	24.44 (25.83)	7.47 (1.37)	1.59 (2.23)
Yes	381.11 (264.20)	16.25 (8.90)	23.67 (15.87)	1.83 (1.56)	67.58 (27.44)	30.68 (31.45)	7.79 (0.96)	1.82 (1.93)
**BDI score**								
Minimal	250.77 (230.13)[a]	9.90 (8.22)[a]	26.12 (21.36)	1.77 (2.33)	47.21 (33.89)[a]	16.25 (20.46)[a]	7.04 (2.01)[a]	1.39 (2.45)
Mild	384.89 (285.90)[a]	16.03 (10.13)[a]	23.41 (11.82)	1.41 (1.08)	59.54 (30.16)[a]	27.73 (32.09)[a]	7.40 (1.26)[a]	1.55 (1.81)
Moderate	376.58 (315.71)[a]	15.46 (9.23)[a]	22.49 (14.36)	1.57 (1.37)	61.78 (28.54)[a]	26.25 (25.67)[a]	7.67 (1.08)[a]	1.57 (2.18)
Severe	341.28 (226.75)[a]	16.21 (8.54)[a]	22.08 (12.82)	1.87 (1.32)	60.15 (24.26)[a]	30.45 (27.17)[a]	7.82 (0.58)[a]	1.93 (2.23)
**IT confidence**								
Average	363.26 (333.23)	16.08 (9.85)	22.34 (15.53)	1.35 (1.35)	53.95 (28.97)	22.28 (25.84)	7.42 (1.38)	1.33 (1.94)
Confident	339.89 (248.72)	14.51 (8.85)	24.27 (16.26)	1.58 (1.60)	56.76 (30.04)	24.22 (26.36)	7.64 (1.35)	1.58 (2.39)
Very confident	330.67 (272.97)	13.85 (9.43)	23.17 (15.12)	1.82 (1.66)	59.24 (29.57)	26.98 (27.19)	7.45 (1.30)	1.74 (2.14)

[a]Significant differences between groups in that specific category: $p < .05$
